# Guidelines for Recommended Footwear for Healthy Children and Adolescents: A Rapid Scoping Review to Characterise the Nature and Extent of Footwear Research and Clinical Policy Guidelines

**DOI:** 10.3390/healthcare13131578

**Published:** 2025-07-01

**Authors:** Liam Hughes, Mark I. Johnson, Nic Perrem, Peter Francis

**Affiliations:** 1 The *EVOLVE* Research Group, Department of Health and Sport Science, Southeast Technological University (SETU), Carlow Campus, R93 V960 Carlow, Ireland; peter.francis@setu.ie; 2 Centre for Pain Research, School of Health, Leeds Beckett University, Leeds LS1 3HE, UK; m.johnson@leedsbeckett.ac.uk; 3 NHS Devon, Exeter EX2 5DW, UK; nic.perrem@nhs.net

**Keywords:** paediatric foot development, shoe fit, toe box allowance, footwear design, clinical practice guidelines, minimal footwear, evidence-based recommendations

## Abstract

**Background/Objectives**: Clinical guidelines for children’s footwear vary widely across governmental and clinical sources, reflecting inconsistencies in best practices for paediatric foot health. These discrepancies arise from differing research interpretations, regional priorities, and clinical expertise. This scoping review evaluates existing guidelines and examines the evidence supporting them. The objective of this scoping review was to identify and map existing footwear guidelines for healthy children and adolescents across governmental, professional, and clinical sources, and to evaluate the type and strength of evidence underpinning these recommendations. **Methods**: A systematic search of PubMed, Google Scholar, ScienceDirect, and governmental databases was conducted. Studies on footwear recommendations for healthy children aged 18 months to 18 years were included. Articles published between 1970 and 2024 were considered, as 1970 marked the first mass marketing of running shoes/trainers. **Results**: Footwear guidelines lack standardisation, with variations in definitions, recommendations, and supporting evidence. Key inconsistencies exist in parameters such as fit, flexibility, and toe allowance, with most recommendations based on expert opinion rather than empirical data. Discrepancies in commercial footwear sizing further complicate proper fit assessment. **Conclusions**: This is the first comprehensive review of children’s footwear guidelines, integrating governmental, professional body, and clinical recommendations. While there is consensus on the importance of properly fitting shoes, the literature reveals inconsistencies and reliance on expert opinion rather than high-quality research. This review highlights the need for standardised, evidence-based criteria to guide footwear recommendations and serves as a foundation for future research aimed at bridging the gap between research and practice.

## 1. Introduction

The foot plays four crucial roles in bipedal locomotion. These roles include ensuring stability, weight-bearing, generating forward propulsion, and somatosensory proprioception [1]. The footconsists of 26 bones, 33 joints, and more than 100 muscles, ligaments, tendons, blood vessels, nerves, soft tissues, and skin. These components interact together to create a flexible and stable structure, allowing many different actions required for support, movement, and balance [2,3].

Ensuring optimal foot health during childhood is a concern for parents and healthcare practitioners [4,5]. Optimal foot health and development occur when the natural function and shape of the foot are unhindered [6,7,8,9,10].

Defining ill-fitted footwear can be subjective and difficult to quantify, particularly the appropriateness of fit. Ill-fitting footwear may be viewed as footwear that may be too wide, too long, too narrow, or too short for the wearer’s foot [7,11]. However, ill-fitting could also extend beyond these parameters and refer to footwear that hinders the normal functioning of the foot such as cushioning or arch support [11]. Hollander et al. demonstrated that children who were habitually barefoot exhibited significantly higher medial longitudinal arches and lower hallux angles compared to habitually shod children further demonstrating the impact of footwear on foot health [12].

The pre-school era (1–3 years old) is widely regarded as the most crucial phase in terms of foot formation, mostly due to the magnitude of developmental transformations that occur during this time [13,14,15,16]. At birth, ossification centres are visible on radiograph imaging in the cuboid, calcaneus, talus, metatarsals, and phalanges.

The bones of the rearfoot and midfoot mature by enchondral ossification and the cuneiforms begin to ossify during the first year of life. The navicular ossification center generally appears between the ages of ~2–4 years. Secondary ossification centres appear in the metatarsals and phalanges between ~6 and 24 months and fuse during the teen years [17].

The development of arch formation is a continual process, with the longitudinal and transverse arches becoming observable by the age of ~6 years in most children [18,19,20,21]. During this stage of development, the musculature, tendons, ligaments, and connective tissue in children’s feet undergo a strengthening process, leading to enhanced foot stability [22].

Children’s feet go through numerous changes until they reach adulthood. A prospective study suggests children aged between 1 and 2.5 years can require shoe size changes every two to three months. The rate of foot growth slows thereafter and changes in footwear are generally required every 4 to 6-months in 4 to 6-year olds, respectively [23,24] Girls’ feet usually grow an additional 2% in length from ages ~12–17 years, while boys’ feet can grow an additional 10%. There also appear to be different rates of growth in the calcaneus between boys and girls in longitudinal data observing plain radiographs. Calcaneal growth was quicker in boys compared to girls after the age of 12 [25]. In fact, by the age of 18, the mean length of the calcaneus in boys was approximately 1 cm greater than that in girls [26]. The importance of these complex stages of development cannot be understated and underscores the importance of proper footwear selection. However, guidance for parents and practitioners remains inconsistent and lacking in evidence.

The purpose of children’s footwear is to provide surface protection for feet and protect against the elements [26]. This safety enables children to fully interact with the environment and develop their basic motor skills. Due to these changes, it is important that children and adolescents wear the correct footwear [27].

Leung et al. [28] propose a general growth pattern in which foot length in girls increases in a linear manner between the ages of ~4 and ~13, while in males it increases between the ages of ~4 and ~14. In contrast, Liu et al. [27] propose a precise estimate of the cessation of growth in which boys typically stop growing in foot length at around 15 to 16 years, whereas girls reach this point at approximately 13 to 14 years. Typically, the epiphyseal growth plates in the feet are expected to ossify and fully fuse between the ages of 14 to 16 years in females and 16 to 18 years in males. However, this timeline may vary depending on individual genetic factors [29]. Therefore, due to the malleable nature of feet and the time course of foot development, the foot environment can have an impact on foot development [28]. For instance, cross-sectional studies indicate that children who typically wear footwear exhibit a comparatively reduced medial longitudinal arch in comparison to children who regularly engage in barefoot activities. Conversely, it has been determined through prospective research that the development of the medial longitudinal arch occurs naturally and irrespective of footwear [30,31]. These findings were also highlighted in a 2024 meta-analysis by Liu et al. [27]. 

As previously highlighted, feet go through various stages of development during childhood. It is this plasticity that makes the design, size, and shape of footwear important [32]. This argues the need for ensuring children and adolescents are wearing footwear that is well-fitted.

Research in populations that are habitually barefoot has demonstrated that individuals who wore minimal footwear exhibited a stiffer longitudinal arch, thus leading to larger abductor hallucis and abductor digiti minimi muscles when compared to individuals who wore “conventional footwear” [33]. This raises questions such as, which type of footwear may be recommended for developing feet. Therefore, the objective of this rapid scoping review was to identify current footwear guidelines present in the available literature, inclusive of government, professional body, and expert clinical panel guidelines, and assess the evidence to support these guidelines. To our knowledge, there is no comprehensive synthesis that currently exists that maps these guidelines against the available evidence.

## 2. Methods

The techniques used for this review were created in accordance with recommendations made by Arksey and O’Malley [34] Levac et al. [35] and Tricco et al. [36]. The review was structured in line with the PRISMA-ScR framework [36].

The rapid scoping review process was broken down into five iterative steps in the methodological approach: defining the research topic, locating pertinent studies, choosing studies, charting the data, and compiling, summarising, and reporting the findings.

### 2.1. Identifying the Research Question

The main topic for investigation was to identify current footwear guidelines present in the available literature, inclusive of government, professional body, and expert clinical panel guidelines. The secondary aim was to assess whether there is evidence to support these recommendations.

### 2.2. Identifying the Relevant Studies

A: A literature search was performed using Google Scholar, Scopus, ScienceDirect, and PubMed. The search terms were formulated by identifying keywords from the relevant literature and through discussions with an experienced reference librarian. MeSH terms and Boolean search terms were used. The terms employed in the search included (children* OR adolescents* OR paediatric* OR pediatric*) AND (footwear* OR shoes* OR conventional shoes* OR trainers*) AND (recommendations* OR guidelines*). The inclusion criteria allowed for reports published between 1970 and 2024. The year 1970 was chosen due to the first mass marketing of running shoes/trainers, which have since become the most commonly worn type of footwear among children and adolescents.

B: Identifying policy documents. Governmental bodies were contacted via email and phone for copies of their policy guidelines for footwear recommendations for children. The Health and Safety Executive (HSE) in Ireland, the United Kingdom’s National Health Service (NHS), the American Podiatry Medical Association (APMA), and the World Health Organization (WHO) bodies in the United States, Germany, Brazil, India, Russia, and China were contacted.

### 2.3. Study Selection

A: Studies were included in the review if they met the following set of inclusion criteria:

- Included otherwise healthy male and female children and adolescents aged from 18 months to 18 years old.
- Included sports shoes, school shoes, and conventional shoes.
- Published in English.
- Published between January 1970 and December 2024.
- Included peer-reviewed manuscripts such as randomised control trials, prospective studies, cross-sectional observational studies, longitudinal studies, repeated measures trials, Delphi studies, observational reviews, literature reviews, and published clinical guidelines from professional or governmental bodies.

B: Policy selection

Policies were included in the review if they met the same criteria mentioned above. Studies were excluded if they were not in English or included children with foot deformities.

### 2.4. Data Charting

A preliminary assessment of study abstracts was conducted to determine their suitability for inclusion in the review. For studies deemed potentially relevant, the full text was reviewed to confirm their appropriateness based on the established criteria. This process was repeated three times to ensure a thorough and iterative evaluation, refining the selection at each stage with rigor. During the data charting process, we systematically extracted information on key variables associated with footwear recommendations. These included the following:

- Fit—definitions and specific measurement criteria (e.g., millimetre allowances, thumb width);
- Width—methods of classification (e.g., stated width guidelines, WMS system);
- Toe allowance—explicit numerical values or ranges provided;
- Cushioning—presence, absence, or any quantifiable descriptors;
- Flexibility—descriptions related to sole flexibility.

For each variable, we recorded whether the recommendations were supported by empirical evidence, based on expert consensus, or left unspecified. Table 1 summarises the extracted variables and the type of evidence supporting each of the recommendations across the included sources. Levels of evidence were classified according to the Oxford Centre for Evidence-Based Medicine 2009 hierarchy, ranging from Level I (systematic reviews and RCTs) to Level VII (expert opinion).

### 2.5. Collating, Synthesising, and Reporting the Results

**Figure 1:**
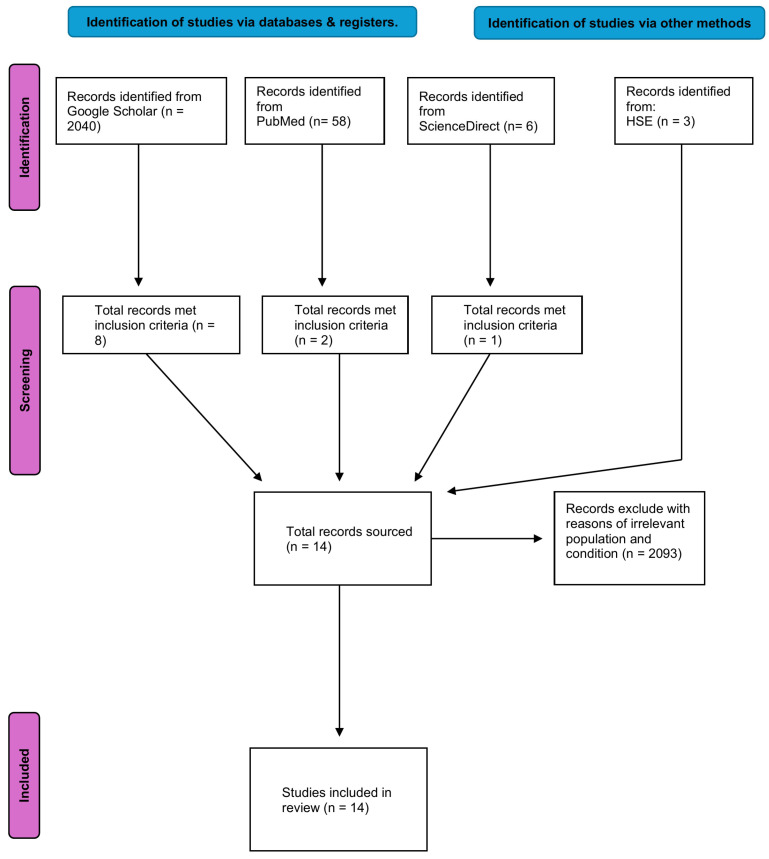
The screening process is diagrammatically represented using a PRISMA-ScR flow diagram, as outlined by Tricco et al. [36].

Data extraction and study selection were conducted by a single reviewer (L.H.) in line with the rapid scoping review methodology, where methodological streamlining is necessary to meet tight timelines. This approach is consistent with best-practice recommendations for rapid reviews [37]. To mitigate potential bias, a second researcher independently spot-checked a subset of the data for accuracy and consistency. This pragmatic approach balanced the need for expediency with efforts to uphold methodological rigor and has been transparently acknowledged as a limitation. The results generated through this review were reported using the PRISMA-ScR tool [34], and the study selection process is illustrated in Figure 1. The narrative report summarises the study findings based on themes identified in the extracted data. The results are given in accordance with the research questions and within the framework of the overall scoping review objectives. Table 2 displays a summary of the papers that are included. The governmental health departments in Russia, China, India, USA, Brazil, and the World Health Organization (WHO) were contacted by email with the research question before the review was underway, but none replied. The governmental health body in Ireland, the HSE, relies on guidance from the UK’s NHS. NHS footwear recommendations are typically based on a combination of local trust protocols, professional podiatry standards (such as those from the Royal College of Podiatry), and condition-specific NICE guidelines, for example in the context of diabetic foot care. However, there is no centralised or universally applied NHS footwear guideline for the general population or for healthy children. Several organisations, including the APMA, have published publicly accessible guidelines aimed at promoting healthy footwear choices for children [38]. A PEO (i.e., population, exposure, and outcome) framework was used (Table 1 below) for the present research [37].

**Table 1:** PEO framework for study eligibility.

Criteria	Determinants
Population	Healthy male and female children and adolescents aged 18 months to 18 years old
Exposure	Footwear, conventional shoes, trainers
Outcomes	Foot recommendations and policy guidelines

## 3. Results

The initial search of reports and studies from databases and registers totalled 2107. Records that were excluded due to irrelevant populations and conditions totalled 2093. A total of 14 articles met the inclusion criteria, which included guidelines from governmental and professional bodies, peer-reviewed reports, and a systematic review. The following sections synthesises these results into five variables: fit, width, toe allowance, cushioning, and flexibility. Each subsection synthesises the findings from the included studies and indicates whether the recommendations were underpinned by empirical evidence or derived from expert consensus.

### 3.1. Fit

Thirteen of the fourteen studies and guidelines included in the review emphasised the significance of “correct fit” in children’s footwear [9,39,40,41,42,43,44,45,46,47,48]. However, only one study—Walther et al. [9]—provided a clear definition using the WMS system. This structured classification system determines fit based on proportional shoe dimensions and the rearfoot-forefoot ratio. Several guidelines recommended allowing space for growth but did not define how “fit” should be assessed, leading to ambiguity. The remaining recommendations were based on expert opinion rather than empirical evidence.

### 3.2. Width

Footwear width was addressed explicitly in only two of the studies. Walther et al. [9] recommended the use of the WMS classification system, which categorizes footwear into small, medium, and wide based on foot width. González Elena et al. [40] proposed a minimum width allowance of 10 mm to ensure proper fit. The remaining sources did not define width parameters; references to width were often mentioned in general terms without measurable criteria or supporting empirical evidence.

### 3.3. Toe Allowance

Out of the fourteen sources, nine provided a specific toe allowance, though there was considerable variation in the reported ranges. González Elena et al. [40] recommended a toe allowance ranging from 5 to 12 mm, whereas Walther et al. [9] advised a fixed distance of 17 mm. Kinz et al. [41] and the Royal College of Podiatry [47] both recommend allowances between 10 and 12 mm. Alfageme-García et al. [49] recommended a toe allowance between 10 and 15 mm. Conversely, Wedge et al. [43] and the Irish Society of Podiatrists [48] employed a less quantifiable approach, recommending an “adult thumb width” as a guideline. These variations underscore the lack of standardisation. Notably, all the recommendations cited were based on expert consensus or practical advice, without reference to supporting empirical data.

### 3.4. Cushioning

Cushioning was the least consistently defined across the included sources. Only three studies—Hillstrom et al. [50], Hollander et al. [12], and Walther et al. [9]—explicitly addressed cushioning. Hollander and Hillstrom both presented empirical data. Hillstrom et al. [50] demonstrated excessive cushioning was associated with altered gait mechanics in early walkers. Walther et al. [9] suggested that the level of cushioning was dependent on the activity type and surface conditions. Notably, none of the included guidelines defined thresholds or offered quantitative criteria to specify adequate cushioning levels.

### 3.5. Flexibility

Thirteen of the fourteen studies and guidelines recommended that children’s footwear should be flexible. Walther et al. [9] and Hillstrom et al. [50] were the only two studies to empirically investigate footwear flexibility, with Hillstrom demonstrating that flexible soles approximate barefoot plantar pressure distribution more effectively. In contrast, the majority of the flexibility-related recommendations lacked precise definitions and were predominantly informed by expert opinion. Alfageme-García et al. [49] recommended that between the ages of 6 months and 3 or 4 years, shoes should be flexible to allow the natural movement of the foot.

### 3.6. Risk of Bias

The risk of bias in individual studies was assessed based on several factors, including the study design, sample size, and the nature of the evidence presented. Many of the included studies relied on expert consensus or opinion rather than high-quality empirical research, which indicates a notable gap in the rigor of the evidence. This reliance on less robust methodologies raises concerns about the validity of the recommendations made in the guidelines. Additionally, the potential for personal bias and “group think” among participants in some studies could have influenced the outcomes. In this review, only one study met criteria as an RCT (Hollander et al., 2014 [12]), and it was assessed using the RoB 2.0 tool Overall, the review highlighted the need for more rigorous methodologies to strengthen the evidence base for footwear guidelines for children and adolescents.

## 4. Discussion

The aim of this rapid scoping review was to identify current footwear guidelines for children and adolescents present in the available literature, inclusive of government, professional bodies, and expert clinical panel guidelines. An in-depth examination of 14 research publications, such as Delphi investigations, systematic reviews, observational studies, and clinical guidelines, provided a thorough insight into the current literature in this field.

The results indicated significant variability in the content and structure of existing footwear recommendations. For example, guidance regarding toe allowance varied from 5 mm to 17 mm across disparate sources [9,39,40,41], while the term “flexibility” was extensively utilised yet rarely defined in quantifiable metrics. Likewise, concepts such as “fit” and “cushioning” were broadly endorsed but infrequently supported by standardised measurement criteria or outcome data. Such inconsistencies underscore an urgent necessity for uniformity in footwear terminology and more explicit methodological foundations across published guidelines. These discrepancies are not merely linguistic but indicate deeper methodological and structural challenges. First, the sources included exhibited considerable variation in design and intent, encompassing Delphi studies [40,41], observational research [42,48], clinical guidelines [43,46,47], and expert opinion articles [45]. This heterogeneity inevitably contributes to variations in evidence quality and the interpretation of footwear variables. Second, commercial interests may also influence certain guidelines, particularly those associated with industry groups or devoid of peer-reviewed substantiation [45]. Lastly, regional and cultural practices are likely to influence variations in footwear norms, with different priorities placed on cushioning, sole rigidity, or durability based on climate conditions, lifestyle choices, or socioeconomic factors.

Significantly, only a minor proportion of the included sources integrated empirical evidence. The majority of guidelines were predicated on expert consensus without transparent references to clinical trials or biomechanical investigations. This was particularly pronounced in recommendations pertaining to toe allowance, flexibility, and fit, which are vital for foot development but are inconsistently supported by data. For instance, Davies et al. [40] and Williams et al. [44] underscored the essential role of suitably fitting footwear in musculoskeletal development, although they provided insufficient operational definitions or empirical verification. Williams [44] further emphasised the inconsistency in commercial sizing standards, highlighting a systemic issue that impacts both clinicians and consumers. In contrast, the systematic review conducted by Alfageme-García et al. [49] offered a structured synthesis of age-specific footwear recommendations grounded in observational and empirical studies. This Level III evidence addressed developmental considerations, providing practical guidance on toe allowance (10–15 mm), sole flexibility, and heel height. Although it did not encompass randomised controlled trials, the review is distinguished by its methodological transparency and developmental specificity, contributing clarity to an otherwise fragmented corpus of literature.

Supplementary evidence supporting the principles of age-appropriate footwear is provided by Walther et al. [9] and Hillstrom et al. [50], both of whom emphasised the significance of flexibility, ventilation, cushioning, and anatomical fit. Walther characterised “fit” through the WMS system, which evaluates foot proportions and alignment to ascertain shoe dimensions. This systematic methodology offers a rare instance of quantitative evaluation within an otherwise qualitative domain. Nonetheless, even Walther did not explicitly delineate cushioning thresholds, acknowledging that these may differ based on individual preferences, types of activities, and environmental surfaces. Hillstrom et al. [50] further demonstrated that excessive cushioning may negatively impact gait biomechanics in early walkers, suggesting that an excess of cushioning is not necessarily beneficial.

Despite these scholarly contributions, the overarching field continues to be predominantly dependent on expert consensus. Guidelines issued by organisations such as the American Podiatric Medical Association [46] and the Royal College of Podiatry [47] generally advocate features such as “flexibility”, “durability”, and “stability”, yet fail to provide operational definitions or reference empirical evidence to substantiate these assertions. For instance, while numerous guidelines advocate for a flexible sole, few delineate how flexibility should be quantified or assessed. This deficiency in precision constrains the applicability of such recommendations in both clinical and manufacturing settings.

**Figure 2:**
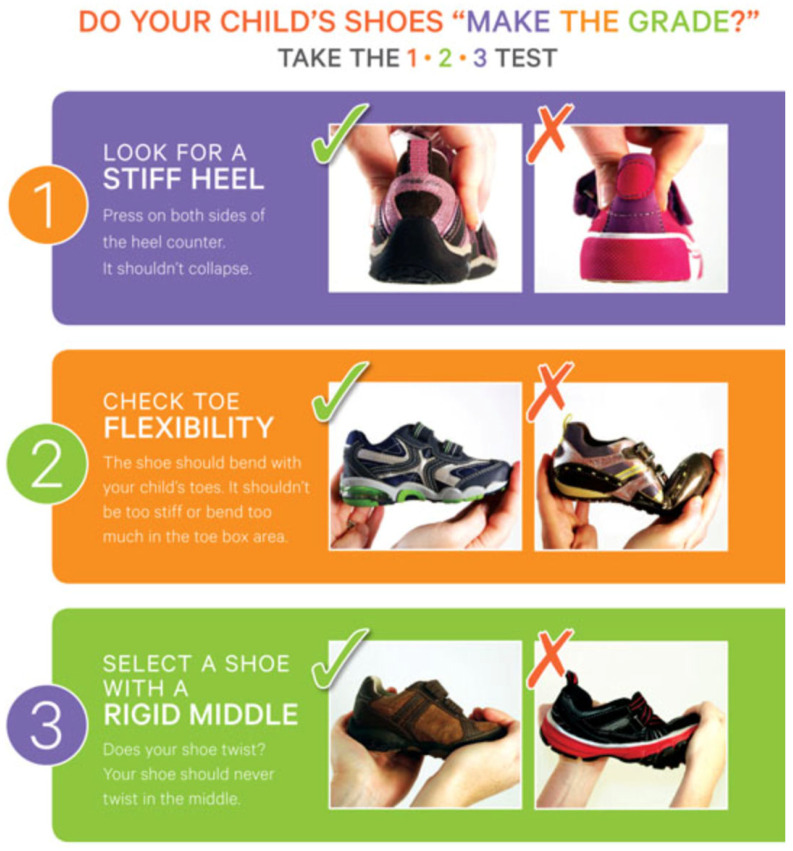
Buying children’s footwear: tips for healthy feet (APMA, 2024 [38]).

An additional concern pertains to the dissemination of information. Numerous organisations offer consumer-orientated resources, such as infographics (Figure 2) [45], but these often oversimplify intricate biomechanical concepts without adequate reference to empirical evidence.

While such materials are accessible and well-intentioned, their scientific underpinnings are frequently ambiguous. For instance, the public-facing recommendations of the American Podiatric Medical Association [46] are easily comprehensible but seldom reference peer-reviewed data, thereby diminishing their utility for clinical application or policy formulation.

The lack of uniform standards and definitions transcends individual footwear characteristics. There exists no consensus on how to measure toe allowance, define an “optimal fit”, or quantify “cushioning.” These deficiencies reflect a dearth of standardisation not only in research methodologies but also in industry practices. As Williams [44] observed, variations in sizing among commercial manufacturers exacerbate the issue, rendering it challenging for practitioners and parents to make informed choices. The review conducted by Alfageme-García et al. [49] reiterated these concerns and advocated for harmonised, evidence-informed standards within the footwear industry.

Biomechanical ramifications of footwear selections are also addressed inconsistently. Tong and Kong [42], alongside Hollander et al. [12], documented that footwear design can substantially influence foot development and gait mechanics, including susceptibility to conditions such as pes planus (flat feet). These investigations provide invaluable empirical insights, suggesting that certain design attributes may predispose children to musculoskeletal dysfunction or injuries, particularly in high-impact scenarios. Such findings highlight the necessity of anchoring footwear recommendations in biomechanical and developmental sciences.

Collectively, the body of literature reveals a troubling dependence on low-level evidence and anecdotal recommendations. While certain guidelines are based on expert consensus and clinical experience, the absence of high-quality empirical data restricts their validity and reproducibility. This issue is particularly problematic in light of the increasing interest in preventive foot health and the escalating commercialisation of children’s footwear.

Parents, healthcare professionals, and educational practitioners necessitate more precise, evidence-driven recommendations that are developmentally suitable and adaptable to various cultural and environmental frameworks.

Future investigations should emphasise the formulation of standardised definitions and validated assessment instruments for footwear characteristics such as flexibility, fit, and cushioning. Moreover, efforts must be made to unify longitudinal and interventional study frameworks to effectively gauge the long-lasting consequences of footwear on the musculoskeletal health of young individuals. There exists an urgent necessity for enhanced transparency in the formulation of footwear guidelines, particularly in terms of revealing conflicts of interest and the evidentiary sources utilised.

In conclusion, while there exists a broad consensus regarding the significance of suitable footwear for children and adolescents, existing guidelines exhibit considerable variability and frequently lack empirical substantiation. Methodological discrepancies, commercial influences, and contextual variability all contribute to this fragmentation. An unmistakable demand is present for standardised, evidence-informed suggestions that can steer both healthcare actions and buyer judgements. This review underscores the necessity of addressing these deficiencies to enhance the clarity, consistency, and applicability of children’s footwear guidelines in the future.

## 5. Limitations

This rapid scoping review is constrained by several limitations. First, although efforts were undertaken to guarantee comprehensiveness, the scope was restricted to English-language studies, thereby potentially omitting pertinent non-English research. In addition, a significant portion of the literature included was based on expert consensus rather than empirical data, which may limit the generalisability and scientific robustness of the findings. Furthermore, the search strategy did not systematically incorporate specialised databases such as PEDro, which might have yielded additional clinically relevant evidence for paediatric populations. Although certain government health agencies were contacted directly, the limited responses received may have resulted in an underrepresentation of national policy guidelines. Additionally, the categorisation of footwear recommendations (e.g., fit, cushioning, flexibility) was, in many cases, based on interpretative judgement due to the absence of clear definitions, potentially introducing subjectivity during data extraction. Finally, the influence of socioeconomic factors on footwear selection and accessibility was not assessed—an omission that may be relevant to the practical implementation and equity of paediatric footwear guidance.

Although only one reviewer conducted the study selection and data extraction, this approach was taken to facilitate timely delivery, as is standard practice in rapid scoping reviews. While this introduces a potential risk of selection bias, the methodology was transparently reported and aligned with accepted frameworks for conducting rapid evidence syntheses.

## 6. Practical Implications

Despite the predominance of low-level evidence, several consensus-based practices can assist clinical decision-making. Healthcare professionals should prioritise achieving a proper fit—allowing approximately 10–15 mm toe allowance and sufficient width—while advising parents to avoid excessively rigid or cushioned footwear, particularly for early walkers. Footwear should be flexible enough to accommodate natural foot movement, especially in children under four years of age. Clinicians should also be aware of commercial sizing inconsistencies and encourage routine footwear assessments to accommodate growth.

To strengthen future guidance, there is a pressing need for validated, age-specific assessment tools and longitudinal studies evaluating the impact of shoe design on musculoskeletal development. In the interim, recommendations should prioritise minimal interference with natural foot function, until unified, evidence-based standards are established.

## Figures and Tables

**Table 2:** Summary of evidence supporting footwear recommendation variables across included sources.

Source	Fit	Toe Allowance	Width	Flexibility	Cushioning
Walther et al.	Expert opinion	Expert opinion (17 mm)	Expert opinion (WMS system)	Expert opinion	Yes, Expert opinion
González Elena et al.	Expert opinion	Expert opinion (5–12 mm)	Expert opinion (≥10 mm)	Expert opinion	Not specified
Kinz et al.	Expert opinion	Expert opinion (12 mm)	Not specified	Expert opinion	Not specified
Royal College of Podiatry	Expert opinion	Expert opinion (10–12 mm)	Not specified	Expert opinion	Not specified
Canadian Pediatric Society	Expert opinion	Expert opinion (10–12 mm)	Not specified	Expert opinion	Not specified
Wedge et al.	Expert opinion	Expert opinion (thumb width)	Not specified	Expert opinion	Not specified
Irish Society of Podiatrists	Expert opinion	Expert opinion (thumb width)	Not specified	Expert opinion	Not specified
Hollander et al.	Empirical	Not specified	Not specified	Empirical	Yes, Empirical
Williams et al.	Expert opinion	Not specified	Not specified	Expert opinion	Not specified
Davies et al.	Expert opinion	Not specified	Not specified	Expert opinion	Not specified
Hillstrom et al.	Empirical	Not specified	Not specified	Empirical	Yes, Empirical
American Podiatric Med. Assoc.	Expert opinion	Not specified	Not specified	Expert opinion	Not specified
Tong and Kong	Empirical	Not specified	Not specified	Not specified	Not specified
Alfageme-García et al.	Empirical	Not specified	Not specified	Empirical (age-specific recommendations)	Yes, Empirical

Note: “Yes, empirical” indicates support from observational or biomechanical study. WMS = Width Measurement System.

## Data Availability

Not applicable.

## References

[1] Eils E., Behrens S., Mers O., Thorwesten L., Völker K., Rosenbaum D. (2004). Reduced plantar sensation causes a cautious walking pattern. Gait Posture.

[2] Tomassoni D., Traini E., Amenta F. (2014). Gender and age related differences in foot morphology. Maturitas.

[3] Ma X., Luximon Y., Luximon A. (2013). Foot structure and anatomy. The Science of Footwear.

[4] Carli A., Saran N., Kruijt J., Alam N., Hamdy R. (2012). Physiological referrals for paediatric musculoskeletal complaints: A costly problem that needs to be addressed. Paediatr. Child Health.

[5] Hodgson L., Growcott C., Williams A.E., Nester C.J., Morrison S.C. (2020). First steps: Parent health behaviours related to children’s foot health. J. Child Health Care.

[6] Staheli L.T. (1991). Shoes for Children: A Review. Pediatrics.

[7] Witana C.P., Feng J., Goonetilleke R.S. (2004). Dimensional differences for evaluating the quality of footwear fit. Ergonomics.

[8] Thompson A.L.T., Zipfel B. (2005). The unshod child into womanhood—Forefoot morphology in two populations. Foot.

[9] Walther M., Herold D., Sinderhauf A., Morrison R. (2008). Children sport shoes—A systematic review of current literature. Foot Ankle Surg..

[10] D’AoÛt K., Pataky T.C., De Clercq D., Aerts P. (2009). The effects of habitual footwear use: Foot shape and function in native barefoot walkers^†^. Footwear Sci..

[11] Branthwaite H., Chockalingam N. (2019). Everyday footwear: An overview of what we know and what we should know on ill-fitting footwear and associated pain and pathology. Foot.

[12] Hollander K., Riebe D., Campe S., Braumann K.M., Zech A. (2014). Effects of footwear on treadmill running biomechanics in preadolescent children. Gait Posture.

[13] Matsuda S., Demura S., Kasuga K., Sugiura H. (2012). Reliability and Sex Differences in the Foot Pressure Load Balance Test and Its Relationship to Physical Characteristics in Preschool Children. Adv. Phys. Educ..

[14] Yurt Y., Sener G., Yakut Y. (2014). Footwear suitability in Turkish preschool-aged children. Prosthet. Orthot. Int..

[15] Vrdoljak O., Kujundžić Tiljak M., Čimić M. (2017). Anthropometric measurements of foot length and shape in children 2 to 7 years of age. Period. Biol..

[16] Harty M.P. (2001). Imaging of Pediatric Foot Disorders. Radiol. Clin. N. Am..

[17] Jimenez-Ormeño E., Aguado X., Delgado-Abellan L., Mecerreyes L., Alegre L.M. (2011). Changes in Footprint with Resistance Exercise. Int. J. Sports Med..

[18] Jiménez-Ormeño E., Aguado X., Delgado-Abellán L., Mecerreyes L., Alegre L.M. (2013). Foot morphology in normal-weight, overweight, and obese schoolchildren. Eur. J. Pediatr..

[19] Barisch-Fritz B., Plank C., Grau S. (2016). Evaluation of the rule-of-thumb: Calculation of the toe allowance for developing feet. Footwear Sci..

[20] Carr J.B., Yang S., Lather L.A. (2016). Pediatric Pes Planus: A State-of-the-Art Review. Pediatrics.

[21] Zhang D., Wang Y. (2017). Study on the Influencing Factors on Comfort of Children’s Shoes. J. Bus. Adm. Res..

[22] Wenger D.R., Mauldin D., Morgan D., Sobol M.G., Pennebaker M., Thaler R. (1983). Foot growth rate in children age one to six years. Foot Ankle.

[23] Gould N., Moreland M., Trevino S., Alvarez R., Fenwick J., Bach N. (1990). Foot Growth in Children Age One to Five Years. Foot Ankle.

[24] Parikh S.N., Weesner M., Welge J. (2012). Postnatal growth of the calcaneus does not simulate growth of the foot. J. Pediatr. Orthop..

[25] Morrison S.C., Price C., McClymont J., Nester C. (2018). Big issues for small feet: Developmental, biomechanical and clinical narratives on children’s footwear. J. Foot Ankle Res..

[26] Wang Y. (2023). [PDF] Understanding the Role of Children’s Footwear on Children’s Feet and Gait Development: A Systematic Scoping Review. Semantic Scholar. https://www.semanticscholar.org/paper/Understanding-the-Role-of-Children%E2%80%99s-Footwear-on-A-Wang-Jiang/397e77da068706bf2955ab3f5707a20dff2f110c.

[27] Liu K.M., Shinoda K., Akiyoshi T., Watanabe H. (1998). Longitudinal analysis of adolescent growth of foot length and stature of children living in Ogi area of Japan: A 12 years data. Z. Morphol. Anthropol..

[28] Leung A., Otley A., Canadian Paediatric Society N and GC (2009). Footwear for children. Paediatr. Child Health.

[29] Echarri J.J., Forriol F. (2003). The development in footprint morphology in 1851 Congolese children from urban and rural areas, and the relationship between this and wearing shoes. J. Pediatr. Orthop. B.

[30] Wenger D.R., Mauldin D., Speck G., Morgan D., Lieber R.L. (1989). Corrective shoes and inserts as treatment for flexible flatfoot in infants and children. J. Bone Jt. Surg. Am..

[31] Kirtley C. (2006). Clinical Gait Analysis: Theory and Practice.

[32] LeVeau B.F., Bernhardt D.B. (1984). Developmental biomechanics. Effect of forces on the growth, development, and maintenance of the human body. Phys. Ther..

[33] Holowka N., Wallace I., Lieberman D. (2018). Foot strength and stiffness are related to footwear use in a comparison of minimally- vs. conventionally-shod populations. Sci. Rep..

[34] Arksey H., O’Malley L. (2005). Scoping studies: Towards a methodological framework. Int. J. Soc. Res. Methodol..

[35] Levac D., Colquhoun H., O’Brien K.K. (2010). Scoping studies: Advancing the methodology. Implement. Sci..

[36] Tricco A.C., Lillie E., Zarin W., O’Brien K., Colquhoun H., Kastner M., Levac D., Ng C., Sharpe J.P., Wilson K. (2016). A scoping review on the conduct and reporting of scoping reviews. BMC Med. Res. Methodol..

[37] Schardt C., Adams M.B., Owens T., Keitz S., Fontelo P. (2007). Utilization of the PICO framework to improve searching PubMed for clinical questions. BMC Med. Inform. Decis. Mak..

[38] Buying Children’s Footwear|Tips for Healthy Feet|Patients|APMA. https://www.apma.org/childrensfootwear.

[39] Davies N., Branthwaite H., Chockalingam N. (2015). Where should a school shoe provide flexibility and support for the asymptomatic 6- to 10-year-olds and on what information is this based? A Delphi yielded consensus. Prosthet. Orthot. Int..

[40] González Elena M.L., Córdoba-Fernández A. (2019). Footwear fit in schoolchildren of southern Spain: A population study. BMC Musculoskelet. Disord..

[41] Kinz W., Groll-Knapp E., Kundi M. (2021). Hallux valgus in pre-school-aged children: The effects of too-short shoes on the hallux angle and the effects of going barefoot on podiatric health. Footwear Sci..

[42] Tong J.W.K., Kong P.W. (2016). Medial Longitudinal Arch Development of Children Aged 7 to 9 Years: Longitudinal Investigation. Phys. Ther..

[43] Wedge J.H. (1985). Assessing Children’s Legs and Feet. Can. Fam. Physician.

[44] Williams C.M., Morrison S.C., Paterson K., Gobbi K., Burton S., Hill M., Kraszewski A.P., Whitney K.A., Scher D.M., Song J. (2022). Young children’s footwear taxonomy: An international Delphi survey of parents, health and footwear industry professionals. PLoS ONE.

[45] Canadian Paediatric Society Protecting and Promoting the Health and Well-Being of Children and Youth|Canadian Paediatric Society 2023. https://cps.ca/en/.

[46] American Podiatric Medical Association (2023). Search|APMA. https://www.apma.org/patients-and-the-public/tips-for-healthy-feet/.

[47] Royal College of Podiatry (2023). College of Podiatry. Royal College of Podiatry Homepage..

[48] SCPI (2023). The Society of Chiropodists & Podiatrists of Ireland. Home..

[49] Alfageme-García P., Hidalgo-Ruiz S., Rico-Martín S., Calderón-García J.F., Jimenez-Cano V.M., Morán-Cortés J.F., Basilio-Fernández B. (2024). Respectful children’s shoes: A systematic review. Children.

[50] Hillstrom H.J., Buckland M.A., Slevin C.M., Hafer J.F., Root L.M., Backus S.I., Kraszewski A.P., Whitney K.A., Scher D.M., Song J. (2013). Effect of shoe flexibility on plantar loading in children learning to walk. J. Am. Podiatr. Med. Assoc..

